# Cognition as a mediator for gait and balance impairments in *GBA*-related Parkinson’s disease

**DOI:** 10.1038/s41531-022-00344-5

**Published:** 2022-06-20

**Authors:** Rosie Morris, Douglas N. Martini, Katrina Ramsey, Valerie E. Kelly, Katrijn Smulders, Amie Hiller, Kathryn A. Chung, Shu-Ching Hu, Cyrus P. Zabetian, Kathleen L. Poston, Ignacio F. Mata, Karen L. Edwards, Jodi Lapidus, Brenna Cholerton, Thomas J. Montine, Joseph F. Quinn, Fay Horak

**Affiliations:** 1 Department of Neurology, Oregon Health and Science University, Portland, OR USA; 2 Department of Sport, Exercise and Rehabilitation, Northumbria University, Newcastle-upon-Tyne, UK; 3 Department of Kinesiology, University of Massachusetts Amherst, Amherst, MA USA; 4 School of Public Health, Oregon Health and Science University, Portland, OR USA; 5 Department of Rehabilitation Medicine, University of Washington School of Medicine, Seattle, WA USA; 6 Sint Maartenskliniek Research Department, Nijmegen, The Netherlands; 7 Portland Veterans Affairs Health Care System, Portland, OR USA; 8 Department of Neurology, University of Washington School of Medicine, Seattle, WA USA; 9 Veterans Affairs Puget Sound Health Care System, Seattle, WA USA; 10 Department of Neurology and Neurological Sciences, Stanford School of Medicine, Palo Alto, CA USA; 11 Lerner Research Institute, Genomic Medicine, Cleveland Clinic Foundation, Cleveland, OH USA; 12 Department of Epidemiology and Biostatistics, University of California, Irvine, CA USA; 13 Department of Pathology, Stanford University School of Medicine, Palo Alto, CA USA

**Keywords:** Parkinson's disease, Parkinson's disease

## Abstract

The extent to which the heterogeneity of gait and balance problems in PD may be explained by genetic variation is unknown. Variants in the glucocerebrosidase (*GBA*) gene are the strongest known genetic risk factor for PD and are associated with greater motor and cognitive severity. However, the impact of *GBA* variants on comprehensive measures of gait and balance and their relationship to cognition remains unknown. We aimed to determine differences in gait and balance impairments in those with and without *GBA* variants (mutation carriers and E326K polymorphism) and explore direct and indirect effects of GBA status on gait, balance, and cognition. 332 participants, 43 of whom had *GBA* variants, were recruited. Participants completed a comprehensive, objective assessment of gait and standing balance using body-worn inertial sensors. Group differences in gait and balance between PD with and without *GBA* variants were assessed with linear regression, adjusting for age, gender, clinical testing site, disease duration, and apolipoprotein E (*APOE)* ɛ4 status. Structural equation modeling (SEM) explored direct relationships between *GBA* status and gait and balance and indirect relationships between *GBA* status and gait and balance via cognition. The *GBA* variant group had more impaired gait (pace and variability) and balance (sway area/jerk and sway velocity), than the non-*GBA* variant group. SEM demonstrated cognition as a mediator of *GBA* status on gait and balance. The close relationships among *GBA*, gait/balance, and cognition suggest potential for novel therapeutics to target the *GBA* pathway and/or cognition to improve mobility in PD GBA variants.

## Introduction

Gait and balance difficulties are cardinal features of Parkinson’s disease (PD), yet these mobility deficits vary greatly across patients. The extent to which the heterogeneity of gait and balance problems in PD may be explained by genetic variation is unknown. The strongest known genetic risk factor for PD is variation within the glucocerebrosidase (*GBA*) gene, which includes the E326K polymorphism and over 200 “pathogenic” mutations responsible for Gaucher disease, although not all variants are reported in PD^1^. Together these variants are present in 7–10% of sporadic PD cases^2^. It is hypothesized that *GBA* mutations result in reduced GCase enzyme activity, which leads to compromised cellular functioning, including increased levels of α-synuclein and Lewy body formation^3,4^.

*GBA* variants are associated with more rapid symptom progression^5,6^, including younger disease onset, faster disease progression, and greater motor severity^7,8^. Furthermore, PD patients who carry *GBA* variants have demonstrated a faster decline in the postural instability and gait difficulty (PIGD) phenotype, as well as poorer dual-task gait speed performance^5,9–11^. However, it is critical to understand the specific gait and balance impairments in people with *GBA-*related PD to provide tailored targets for therapeutics and rehabilitation. To date, there have been no comprehensive comparisons of objective gait and balance measures between PD patients with and without *GBA* variants.

In addition to gait and balance deficits, *GBA* variants are associated with increased severity of non-motor symptoms, including impaired cognitive function^6,12^. A higher incidence of cognitive impairment and dementia is evident in patients with *GBA* variants; in particular, these patients have greater impairment in executive function and visuospatial cognitive domains^6,12^. Furthermore, *GBA* mutations are an independent risk factor for the development of cognitive impairment in PD as well as increased risk of developing dementia with Lewy bodies (DLB)^13,14^. Gait and balance require cognitive input across a number of domains, and those with poorer cognition demonstrate greater deficits in gait and balance measures. Furthermore, a longitudinal relationship has been demonstrated with measures of gait able to predict cognitive decline over three years^15–19^. Due to the known relationship between cognition with gait and balance measures, impairments in gait and balance may be exacerbated in those with *GBA* variants due to the nature of cognitive deficits in this cohort. If poor cognitive function impacts gait and balance function, therapies targeting cognitive function may provide novel treatment routes for this cohort.

**Fig. 1:**
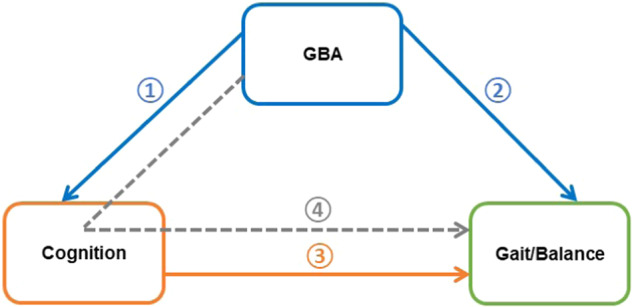
Theoretical model of the impact of GBA on gait and balance. Two pathways are likely to contribute; **①** PD with the GBA variant (GBA+) have poorer cognitive function as previously established^5^ and **②** GBA+ have poorer gait and balance, **③** poorer cognitive function impacts gait and balance function as previously established^15,17,18^. We hypothesize that **④** in GBA+ poorer cognitive function indirectly exacerbates gait and balance impairments via cognition.

The primary aim of this paper was to assess differences in comprehensive, objective measures of gait and balance in people with PD with and without *GBA* variants. A secondary aim was to explore the direct and indirect relationships between *GBA*, cognition, and gait and balance. We hypothesized that people with PD and *GBA* variants would demonstrate poorer gait and balance than those without variants. Further, we hypothesized that *GBA* variants would lead to gait and balance impairments, both directly and indirectly via cognition; this hypothesis is outlined in Fig. 1.

## Results

### Characteristics of participants

A total of 332 participants with idiopathic PD were recruited to the study from the three clinical testing sites. Participant demographic and clinical characteristics are described in Table 1. Of these, 43 participants (13%) carried a pathogenic *GBA* mutation (*n* = 21) or the E326K polymorphism (*n* = 22; see Supplementary Table 1 for specific variants observed^1^). No unclassified nonsense or frameshift mutations were observed in the cohort, and the only other nonsynonymous substitution seen was the T369M polymorphism. While T369M is a risk factor for PD^20^, data from our group suggest that it is not associated with more severe motor or cognitive deficits among PD patients^21^. Therefore, the T369M carriers (*n* = 9) identified in this study were not included in the GBA variant group but were not excluded from analysis. Overall, those with *GBA* variants were more likely to have a higher H&Y score (*p* = 0.028) than those without *GBA* variants, but there was no difference for MDS-UPDRS III or disease duration. Those with *GBA* variants were more likely to have MCI or PDD (*p* < 0.001) than those without *GBA* variants, but there were no other clinical or demographic differences. Within the *GBA* group, those with the E326K polymorphism were older than mutation carriers *(*mutation: 62.2 ± 9.6 years; E326K polymorphism: 69.5 ± 8.3 years, *p* = 0.01), but there were no other demographic or clinical differences observed between the *GBA* subgroups (Supplementary Table 2).

**Table 1:** Demographic and clinical characteristics for those with and without GBA variants.

	No GBA variant (*n* = 289)	GBA variant (*n* = 43)	Difference	*p*
Age (years)	68.2 (8.0)	66.0 (9.6)	−2.2	0.15
Gender: Male, *n* (%)	181 (62.6)	28 (65.1)		0.75
*APOE* ɛ4 carrier, *n* (%)	69 (23.9)	8 (18.6)		0.44
Years of education	16.5 (2.3)	16.5 (2.3)	−0.1	0.88
Ethnicity, *n* (%)				0.99
Asian	6 (2.1)	0 (0)		
Asian, White	1 (0.3)	0 (0)		
Black	1 (0.3)	0 (0)		
Native American, Asian, White	1 (0.3)	0 (0)		
Native American, White	4 (1.4)	0 (0)		
Pacific Islander	1 (0.3)	0 (0)		
Pacific Islander, White	1 (0.3)	0 (0)		
White	268 (93)	43 (100)		
Not reported	6 (2.1)	0 (0)		
MDS-UPDRS III	23.8 (12.0)	27.2 (14.7)	3.4	0.16
Hoehn & Yahr, *n* (%)				**0.028**
1	11 (3.8)	4 (9.3)		
1.5	22 (7.6)	4 (9.3)		
2	192 (66.7)	18 (41.9)		
2.5	45 (15.6)	10 (23.3)		
3	15 (5.2)	6 (14.0)		
4	3 (1.0)	1 (2.3)		
LEDD	638 (481)	744 (466)	106	0.17
Disease duration (years)	7.6 (5.7)	8.0 (5.2)	0.4	0.66
Cognitive status, *n* (%)				
NCI	134 (46.5)	11 (25.6)		**<0.001**
MCI	133 (46.2)	22 (51.2)		
PDD	21 (7.3)	10 (23.3)		

Bold values indicate statistical significance difference.

*MDS-UPDRS III* Movement Disorders Society Unified Parkinson’s disease Rating Scale, *LEDD* Levodopa Equivalent Daily Dose, *NCI* no cognitive impairment, *MCI* mild cognitive impairment, and *PDD* Parkinson’s disease dementia.

Values in the table represent mean (SD) or number (percentage).

### Comparison of gait and balance measures in PD with and without GBA variants

Participants with *GBA* variants had slower gait pace (*β* −0.38, *p* = 0.008) and increased gait variability (*β* 0.53*, p* = 0.002) compared to those without, but there was no difference in gait rhythm or trunk movement during gait between groups, after adjusting for covariates (age, gender, disease duration, apolipoprotein E (*APOE)* ɛ4 status, and clinical testing site). Gait and balance outcomes for those with and without *GBA* variants are compared in Table 2. None of the gait domains differed between those with the *GBA* mutations compared to E326K polymorphism (Supplementary Table 2). Individual gait and balance measures that informed domains are shown in Supplementary Table 3.

**Table 2:** Gait and balance domains between those with and without GBA variants.

	No GBA variant (*N* = 289)	GBA variant (*N* = 43)	Unadjusted differences, standardized			Adjusted differences, standardized^a^		
	Mean (SD)	Mean (SD)	*β*	*p*	95% CI	*β*	*p*	95% CI
Gait								
Pace & turning	0.03 (0.82)	−0.25 (0.93)	−0.28	0.069	−0.58, 0.02	−**0.38**	**0.008**	−0.66, −0.10
Rhythm	−0.04 (0.87)	0.28 (1.37)	0.32	0.151	−0.12, 0.75	0.34	0.117	−0.09, 0.77
Variability	−0.07 (0.79)	0.47 (1.02)	**0.54**	**0.001**	0.21, 0.87	**0.53**	**0.002**	0.19, 0.87
Trunk movement	0.01 (0.74)	−0.05 (0.92)	−0.06	0.699	−0.35, 0.24	−0.09	0.538	−0.38, 0.20
Balance								
Sway area & jerk^b^	−0.06 (0.82)	0.43 (1.33)	**0.50**	**0.017**	0.09, 0.91	**0.50**	**0.011**	0.11, 0.89
Sway velocity^b^	−0.04 (0.80)	0.24 (0.91)	0.27	0.060	−0.01, 0.56	**0.28**	**0.041**	0.01, 0.55
Sway frequency ML	−0.03 (0.89)	0.19 (1.09)	0.22	0.204	−0.12, 0.56	0.22	0.234	−0.14, 0.57
Sway frequency AP	−0.05 (0.86)	0.31 (1.22)	0.36	0.063	−0.02, 0.73	0.37	0.051	0.00, 0.74

Bold values indicate statistical significance difference.

^a^Analysis adjusted for age, gender, disease duration, clinical testing site, and *APOE* ɛ4 group status.

^b^Values log transformed for statistical analysis; mean and SD given on original scale.

Values in the table represent mean (SD).

Participants with *GBA* variants demonstrated greater postural sway area/jerk (*β* 0.50*, p* = 0.011) and greater sway velocity (*β* 0.28*, p* = 0.041) than those without variants, when comparing domains of balance, after adjusting for covariates. None of the balance domains differed between *GBA* variant subgroups; participants with *GBA* mutations had similar balance to participants with the E326K polymorphism (Supplementary Table 2). Individual measures that informed balance domains are shown in Supplementary Table 3.

Additional analysis identified that when controlling for cognitive group, the pace & turning domain and the sway velocity domains were no longer significantly different between groups. However, gait variability and sway area & Jerk remained significantly different (Supplementary Table 4).

### Cognition function in PD with and without GBA variants

Participants with *GBA* variants had a significantly poorer MoCA score (*β* −0.66*, p* = 0.004), worse attention/executive function (TMT B-A; *β* 0.39, *p* = 0.023), worse visuospatial function (JoLO; *β* −0.41*, p* = 0.012), poorer immediate recall (HVLT-R total recall; *β* −0.51, *p* = 0.002) and poorer semantic fluency (Animals; *β* −0.40, *p* = 0.049) compared to those without the *GBA* variant, when adjusting for age, gender, years of education, disease duration, data collection clinical testing site, and *APOE* ɛ4 status. Differences in cognitive performance between groups with versus without *GBA* variants are shown in Table 3. There was no difference in cognitive performance between the *GBA* variant groups (Supplementary Table 2).

**Table 3:** Cognitive performance in those with and without GBA variants.

	No GBA variant (*N* = 289)	GBA variant (*N* = 43)	Unadjusted differences, standardized			Adjusted differences, standardized^a^		
	Mean (SD)	Mean (SD)	*β*	*p*	95% CI	*β*	*p*	95% CI
Global cognition								
MoCA	26.0 (3.0)	23.7 (5.3)	−**0.63**	**0.008**	**−**1.10, −0.16	−**0.66**	**0.004**	−1.10, −0.21
Attention/executive function								
TMT B-A	55 (44)	75 (59)	**0.44**	**0.039**	0.02, 0.85	**0.39**	**0.023**	0.05, 0.73
LNST	9.8 (2.4)	9.4 (2.9)	−0.16	0.422	−0.54, 0.23	−0.17	0.352	−0.52, 0.19
Visuospatia								
JoLO	12.2 (2.3)	11.4 (2.4)	−0.33	0.060	−0.67, 0.01	−**0.41**	**0.012**	−0.72, −0.09
Memory								
HVLT-R total recall	24 (5)	21 (6)	−**2.74**	**0.007**	−0.84, −0.13	−**0.51**	**0.002**	−0.83, −0.19
HVLT- delayed recall	8 (3)	7 (4)	−0.94	0.104	−0.65, 0.06	−0.31	0.068	−0.64, 0.02
Language								
Semantic fluency	20 (6)	18 (7)	−1.82	0.134	−0.69, 0.09	−**0.40**	**0.049**	−0.80, 0.00

Bold values indicate statistical significance difference.

Values in table represent mean (SD).

*MoCA* Montreal Cognitive Assessment, *LNST* Letter Number Sequencing Test, *TMT* Trial Making Test, *JoLO* Judgment of Line Orientation, *HVLT-R* Hopkins Verbal Learning Test-Revised.

^a^Analysis adjusted for age, gender, disease duration, clinical testing site, years of education, and *APOE* ɛ4 status. Bold text indicates significant difference.

### Direct and indirect associations of GBA variants with gait, balance, and cognition

**Fig. 2:**
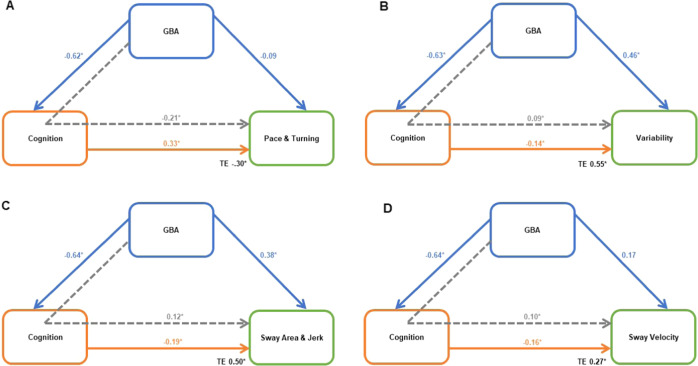
Structural Equation Models (SEM) for both direct and indirect relationships. SEM for **A** pace and turning, **B** gait variability, **C** sway area and Jerk and **D** sway Velocity. Solid arrows indicate direct pathways, dashed arrows indicate indirect pathways. * symbolizes significant standardized *β* value, TE total effect. Cognition is a latent variable including attention/executive function, global cognition, memory, and language.

Significant, direct relationships between *GBA* and cognition (relationship 1) and between cognition and gait (relationship 3) were found for all 4 domains of gait and balance (see Fig. 1). The SEM for the four significant domains of gait and balance are shown in Fig. 2. Standardized regression weights demonstrate the relative strength of associations between the variables in the model. All models were a good-excellent fit as specified by the criteria above. All significant differences in cognitive performance between groups were retained in the latent variable except for the JoLO, which did not meet the loading variable requirement.

The pace/turning domain of gait showed no direct relationship with *GBA* (β −0.09), but there was a significant indirect relationship between *GBA* status and pace/turning via cognition (*β* −0.21; Fig. 2A). The variability domain of gait showed a significant, direct relationship between *GBA* and gait variability (β 0.46) as well as a significant, indirect relationship between *GBA* and gait variability via cognition (*β* 0.09; Fig. 2B).

For the *sway area/jerk* domain of balance, SEM demonstrated a significant direct relationship between *GBA* and sway area/jerk (*β* 0.38) and a significant indirect relationship between *GBA* and sway area/jerk via cognition (*β* 0.12; Fig. 2C). For the sway velocity domain, there was no direct relationship between *GBA* and sway velocity (*β* 0.17) but there was a significant indirect relationship between *GBA* and sway velocity via cognition (*β* 0.10; Fig. 2D).

## Discussion

This study compared comprehensive gait, balance, and cognitive measures in people with and without *GBA* variants in a well-characterized, multi-site PD cohort. In addition, we used SEM to examine the causal relationships among gait/balance and cognition in *GBA*-related PD to determine the extent to which cognitive impairment impacts gait and balance deficits. In our cohort, those with *GBA* variants demonstrated greater impairments in the pace/turning and variability domains of gait and the sway area/jerk and sway velocity domains of balance. Furthermore, our SEM indicated both direct associations between *GBA* status and gait variability and postural sway area/jerk as well as widespread, indirect associations for *GBA* on gait and balance via cognition as a mediator. Although future validation work in independent cohorts is needed, our findings suggest novel avenues for treating mobility impairments in PD.

Previous work demonstrates that GBA carriers have greater motor symptom severity and are more likely to present with the PIGD motor phenotype^5,9^. We identified impairments in specific domains of gait (pace/turning and variability) and balance (sway area/jerk and velocity) in *GBA* carriers compared to those without. In contrast, other domains of gait (rhythm and trunk movement) and balance (sway frequency) did not differ significantly between *GBA* groups. This finding is consistent with the idea of multiple mechanisms underlying the control of different gait and balance domains, as evidenced by inconsistent effects of dopaminergic replacement therapy^22,23^. Our findings support previous evidence indicating the impacts of *GBA* variants on mobility and extend prior work by identifying specific gait and balance domains that are more affected in those with *GBA* variants, even when accounting for disease duration.

*GBA* carriers with PD have greater accumulation of α-synuclein due to the reduced expression of the GCase enzyme^2,13^. As poorer gait and balance have been noted in mouse models overexpressing α-synuclein^24^, this may indicate that α-synuclein accumulation in non-dopaminergic pathways underlies the gait and balance deficits shown in *GBA* carriers^25^. Importantly, reduced GCase activity occurs not only in *GBA*-related PD, but also idiopathic PD^26^, suggesting a novel treatment target for mobility deficits in PD.

Gait and balance dysfunction are associated with cognitive dysfunction in the elderly, with specific associations for cognitive domains related to distinct measures of gait and balance^18,27^. We, therefore, hypothesized that cognition would impact gait and balance in our PD cohort and that the impact of cognitive impairment would be exacerbated in GBA carriers. Our SEM confirmed that cognitive impairment was significantly associated with specific gait and balance deficits^15,17^ and that *GBA* status was significantly associated with poorer cognition^6^.

Cognitive status mediated an indirect relationship for all gait and balance domains that were worse in those with *GBA* variants compared to those without. No direct effect of *GBA* was found for the pace/turning domains of gait or the sway velocity domain of balance, but an indirect effect via cognition was found for both. In contrast, gait variability and sway area showed both a direct effect of *GBA* as well as an indirect effect via cognition. This indicates that cognition, in addition to other *GBA*-related factors, likely contributes to gait and balance impairments in people with PD.

Our results support the notion that *GBA* variants impact gait and balance deficits in various ways. First, measures such as gait speed and turning speed are associated with cognition in aging and PD populations^15,16^, indicating potential dysfunction in cortical areas associated with cognition. Second, measures such as gait variability and sway area may be impacted by the effects of *GBA* variants on other regions or circuits that are less dependent on cognitive control, such as subcortical areas^18^. Further work is needed to inform this hypothesis, including replication in an independent cohort, imaging studies, and longitudinal analysis to determine the trajectory of mobility and cognitive impairments in *GBA* variants.

The impact of *GBA* variants occurs on a continuum, where severe pathogenic mutations are known to impact motor and cognitive signs to a greater extent than mild mutations^28,29^. The severity of specific variants in our cohort is summarized in Supplementary Table 1, with roughly equal numbers of mild and severe mutations. However, we found no differences in gait, balance, or cognitive characteristics between those with mild versus severe mutations (Supplementary Table 5). These findings are surprising considering the known continuum of mutation severity on symptoms. Heterogeneity may have impacted our findings due to the small numbers in our cohort, and further work should explore the impact of mutation severity on gait, balance, and cognition.

Our findings are consistent with the idea that *GBA* status is associated with specific gait and balance deficits both directly and indirectly, via cognitive impairments. Although our work needs to be replicated in future cohorts, it supports a precision-medicine approach for both rehabilitative and pharmacological interventions^30^. In the future, rapid and cost-effective methods will emerge to identify people with PD who have *GBA* variants, which could enable referral to physical therapy to address balance and gait impairments even before their earliest presentation^31^. Exercise programs targeting cognitive function as well as balance and gait at an early disease stage may improve mobility and prolong the time to first fall^30^. Furthermore, targeted medications to increase GCase enzyme activity may improve cognition and mobility^13,32^. A recent study assessing efficacy for ambroxol therapy to increase GCase activity demonstrated that the treatment was safe, well-tolerated, and able to penetrate the blood-brain barrier^33^, but the impact on mobility is yet to be assessed. Furthermore, treadmill training in mice expressing human α-synuclein improved gait (specifically, speed and stride length) and balance, demonstrating the importance of exercise, in addition to dopaminergic medications, to target mobility impairments^24^.

This study has several limitations. First, our cohort of *GBA* carriers was relatively small compared to those without *GBA*. Recruitment of *GBA* carriers is challenging, as they account for only 7–10% of patients with PD. We also pooled patients with pathogenic mutations and the E326K polymorphism for this analysis. Our results and other studies^5,6^ demonstrate similarities in motor and cognitive functions in these groups, providing justification for this grouping. Second, it is possible that some people with PD in our cohort were unable to complete gait and balance assessments; for example, if they were unable to stand unsupported for 30 seconds. Therefore, PD participants with *GBA* variants included in this study may not capture the most severely affected participants, which may bias results. Third, we did not control for multiple comparisons in our analysis. This decision was made due to the exploratory nature of our study, the first of its kind in this rare genetic cohort with well characterized gait, balance, and cognition. We sought to limit the impact of multiple comparisons by using independent domains of gait and balance rather than individual characteristics^34^, however, our exploratory results should be treated with caution until they are replicated in independent cohorts. Fourth, we used disease duration rather than disease severity in our statistical modeling as we sought to determine how cognition impacts gait and balance impairment, irrespective of disease severity. Fifth, our cohort was also enriched for PD *APOE* ε4 carriers. This may have impacted our findings, but separate analyses demonstrated that differences in gait and balance characteristics between those with and without the GBA variant remained even when *APOE* ε4 carriers were removed. Finally, our analysis was cross-sectional, but longitudinal studies of gait and balance in GBA cohorts could inform our understanding of the physiological mechanisms for how GBA variants affect gait and balance.

This study demonstrated that among people with PD, specific domains of gait (pace/turning and variability) and balance (sway area/jerk and velocity) were more affected in those carrying *GBA* variants compared to those without. In addition, cognitive impairments impacted the relationships between *GBA* and gait and balance. These findings have implications for personalized medicine to improve mobility among people with PD.

## Methods

### Participants

Participants with PD were recruited from the Pacific Udall Center (PUC) Clinical Core, comprised of three clinical testing sites: Oregon Health and Science University/Portland VA Medical Center, Portland, OR; University of Washington/VA Puget Sound Health Care System, Seattle, WA; and Stanford University, Palo Alto, CA. All subjects provided informed written consent approved by the joint Institutional Review Boards at Oregon Health & Science University, the VA Portland Health Care System, University of Washington, VA Puget Sound Health Care System, and Stanford University, (Stanford University, IRB- 37967). Participants were included in the study if they: (i) met the United Kingdom Parkinson’s Disease Society Brain Bank (UKBB) clinical diagnostic criteria for PD, with the modification that having more than one affected relative was not an exclusion criterion^35^, (ii) had no history of other neurological disorders that affected cognition, e.g., large-vessel stroke or severe traumatic brain injury, and iii) were able to stand unsupported for a minimum of 30 seconds. Participants who were taking medication were tested ‘on’ PD medication for all assessments. Because the overall goal of this study was to examine balance and gait measures in genetic subgroups of PD, the study cohort was enriched in both *GBA* variants and *APOE* ε4 carriers by selectively inviting every eligible *GBA* and *APOE* ε4 carrier in the PUC Clinical Core to participate in the study. *APOE* ε4 carriers have a greater risk of cognitive decline and dementia^36^ and therefore this genetic type has been controlled for in statistical analyses.

### Demographic and clinical characteristics

Age, gender, height, and years of education were recorded for all participants. PD motor severity was assessed using the Movement Disorders Society Unified Parkinson’s Disease Rating Scale (MDS-UPDRS III)^37^ and the modified Hoehn and Yahr scale (H&Y)^38^. Daily dopamine replacement therapy dose was calculated using the levodopa equivalent daily dose score (LEDD)^39^. Each participant was assigned a diagnosis of no cognitive impairment (NCI), mild cognitive impairment (MCI), or Parkinson’s disease dementia (PDD). The diagnosis was established at biweekly diagnostic consensus conference between all clinical testing sites in accordance with specific criteria^40^.

### Genotyping

The *GBA* group within this study included both low frequency mutations that cause Gaucher disease and the E326K polymorphism which occurs at a frequency >1% in the population. Hereafter, they will be referred to together as “*GBA* variants”. Genomic DNA was extracted from peripheral blood or saliva samples using standard procedures. The entire *GBA* coding region was screened in every participant using Sanger sequencing to capture all known pathogenic mutations (defined as those reported in patients with Gaucher disease^1,41^), the E326K polymorphism (rs2230288), and other potentially relevant variants. All sequencing was performed at a single laboratory at the PUC site in Seattle using methods previously described^6^.

By design, our cohort was enriched for *APOE* ε4 carriers. *APOE* ε4 status was determined by genotyping the *APOE* single nucleotide polymorphisms rs429358 and rs7412 (which define the ε2, ε3, and ε4 alleles), using TaqMan Assays as previously described^42^.

### Gait and balance assessment

Participants performed a standardized instrumented gait and balance assessment wearing six inertial sensors (Opals by APDM Wearable Technology of ERT, Portland, OR). Inertial sensors were attached with elastic Velcro straps to the wrists and feet, as well as on the sternum and fifth lumbar vertebrae. To assess gait, participants were asked to walk at their normal pace back and forth on a straight 7 m walkway in a quiet hallway for 2 min. To assess balance, participants stood quietly, looking an image straight ahead for 60 seconds with their arms at their sides. At the start of the gait and balance trials, a template was used to achieve consistent foot placement (10 cm between left and right heel and 30° outward rotation of the feet).

### Gait and balance measures

A total of 15 gait and 13 balance measures were analyzed from the inertial sensors. To reduce variables for statistical analysis but maintain a wide representation of measures, gait and balance measures were allocated to independent domains based on our previous model^18^. The 4 domains of gait were pace/turning, rhythm, variability, and trunk movement. The four domains of balance were sway area/jerk, sway velocity, sway frequency anteroposterior (AP), and sway frequency medio-lateral (ML).

### Cognitive assessment

Study visits included a comprehensive assessment of cognition^40^. To assess different domains of cognition, the MoCA was selected to assess *global cognition*, the Trail Making Test (TMT) B-A and the letter number sequencing test (LNST) were selected to assess *executive function*, the Hopkins Verbal Learning Test-Revised (HVLT-R) Total recall and Delayed recall were selected to assess *memory* (immediate and delayed recall respectively). The Judgment of Line Orientation (JoLO) test assessed *visuospatial function* and language was assessed via *semantic fluency* (Animals).

### Data analysis

Distributions of all metrics were inspected using boxplots, histograms, and normal quantile plots. Variables that were positively skewed were transformed (natural log 10) prior to further analysis to better meet modeling assumptions. Domain scores were calculated for the 8 gait and balance domains, with groupings based on a previous principal component analysis^18^. Domain scores were calculated by standardizing the component measures (using the value minus the mean, divided by the standard deviation), multiplying by −1 to reverse scaling if needed for consistency, and taking the average (no weighting was used). Demographic and clinical characteristics were compared between PD, with and without *GBA* variants, using Student’s t-tests with unequal variances and χ^2^ tests. To ensure *GBA* subtypes (*GBA* mutation and E326K polymorphism) could be grouped, differences between *GBA* variant subtypes for demographic, clinical, gait, balance, and cognitive data were compared using independent Student’s t-tests.

### Comparing gait and balance in GBA and non-GBA related PD

We compared group mean differences in individual gait and balance domain scores between PD with and without *GBA* variants using generalized linear models (GLM) and applying a robust estimator with (1) an indicator variable for *GBA* variants and (2) covariate variables. Covariates included age, gender, clinical testing site, and *APOE* ɛ4 status (due to high percentage of *APOE* ɛ4 carriers within our sample). In addition, disease duration was included as a covariate as a proxy for disease severity, as measures such the MDS-UPDRS III and H&Y directly relate to our primary outcome measure of gait and balance. Additional analysis was performed controlling for cognitive group (NCI, MCI, or PDD) to decipher whether results were driven by *GBA* variant status or cognitive status.

This is study is unique in having well-characterized gait, balance, and cognition measures in a cohort enriched for rare *GBA* variants. Due to the exploratory nature of this work, a *p*-value <0.05 in the adjusted model was considered evidence of association for the next stage of analysis. Gait and balance domains significantly related to the *GBA* variant were taken forward to the SEM analysis.

### Exploring the direct and indirect relationships between GBA, cognition, and gait and balance

To explore the direct and indirect effects of cognition on gait and balance, we first sought to identify differences in cognition between those with and without *GBA* variants. Differences were assessed using a GLM with a robust estimator with the indicator variable for *GBA* variants and adjusting for age, gender, disease duration, clinical testing site, *APOE* ɛ4 status, and years of education. A *p*-value below 0.05 in the adjusted model was considered evidence of association with those related to the *GBA* variant taken forward to the SEM analysis.

To explore the relationship between *GBA* variants, cognition, and gait and balance impairments, SEM was used to estimate direct and indirect relationships presented in Fig. 1. We sought to explore direct relationships between *GBA* and cognition (Relationship 1, Fig. 1), between *GBA* and gait/balance (Relationship 2, Fig. 1) and between cognition and gait/balance (Relationship 3, Fig. 1). We then sought to explore the indirect relationship between *GBA* and gait/balance via cognition (Relationship 4, Fig. 1).

Models were examined only for the balance and gait domains that were significantly different between genetic groups as determined in the first stage of analysis. Each model included *GBA* group and a single domain of gait or balance, with cognition entered as a latent variable (using standardized scores for each cognitive test). Cognitive tests that differed between *GBA* and non-*GBA* groups at a significance threshold of *p* < 0.05 were entered onto the latent variable. Second, latent variable loadings were examined to determine if any cognitive tests had a poor variable loading (variable loading <0.60) as these were then removed from the model. Maximum likelihood methods were used to estimate parameters using all available data. The goodness of fit were assessed using 1) *X*^2^ test for the model versus a saturated model did not reject the null at *p* < 0.05, 2) comparative fit index (CFI) > 0.90, 3) root mean squared error of approximation (RMSEA) at <0.08, 4) Akaike’s information criterion and Bayesian information criterion lower than for other formulations with the same variables^43,44^. SPSS version 24 and Stata version 15 were used for data analysis.

## Supplementary information

Supplementary Material

## Data Availability

The data that support the findings of this study are available from the corresponding author upon reasonable request.
