# Supplementary material for: Cognition as a mediator for gait and balance impairments in GBA-related Parkinson’s disease
Source: NPJ Parkinsons Dis. 2022 Jun 20;8:78. doi: 10.1038/s41531-022-00344-5 (PMC9209443; doi:10.1038/s41531-022-00344-5)
Supplement: Supplementary file 1 — Supplementary Material [file 41531_2022_344_MOESM1_ESM.pdf]

Nucleotide change	Amino acid change	n	Functional Class	Severity
c.1226A>G	p.N370S	11	Missense	Mild
c.1448T>C	p.L444P	5	Missense	Severe
c.84dupG	p.L29Afs	3	Frameshift	Null
IVS2+1G>A	--	1	Splice site	Null
c.1504C>T	R463C	1	Missense	Severe

Supplementary Table 1. Pathogenic *GBA* variants observed.[1] *A mutation was considered "pathogenic" if it was previously reported in at least one patient with Gaucher disease in the homozygous or compound heterozygous state or if it was predicted to have a clearly deleterious effect on function (e.g., frameshift or nonsense mutations).*

Supplementary Table 2. Demographic, clinical and cognitive characteristics for GBA mutation carriers and GBA E326K Polymorphism variants. Values in table represent Mean (SD) or number (percentage).

		GBA Mutation Carrier (N=21)	GBA E326K Polymorphism (N=22)	*p*
**Demographics**	**Age (years)**	62.2 (9.6)	69.5 (8.3)	**0.01**
	**Gender M/F (%)**	11/10 (52%/48%)	17/5 (77%/33%)	0.09
	**Years of education**	16.8 (2.3)	16.2 (2.3)	0.38
	**MDS-UPDRS III**	26.2 (15.3)	28.1 (14.5)	0.68
	**Hoehn & Yahr n (%)**			0.21
	**1**	3 (14%)	1 (5%)	
	**1.5**	1 (5%)	3 (14%)	
	**2**	9 (42%)	9 (40%)	
	**2.5**	6 (29%)	4 (18%)	
	**3**	1 (5%)	5 (23%)	
	**4**	1 (5%)	0 (0%)	
	**LEDD**	833 (517)	659 (407)	0.23
	**Disease duration (years)**	9.2 (5.2)	6.9 (5.0)	0.16
	**Cognitive status**			0.42
	**NCI**	7 (33%)	4 (18%)	
	**MCI**	10 (48%)	12 (55%)	
	**PDD**	4 (19%)	6 (27%)	
**Cognition**	**MoCA**	24.7 (4.9)	22.8 (5.6)	0.25
	**TMT B-A**	78 (51)	72 (68)	0.79
	**LNST**	9.8 (3.3)	9.0 (2.5)	0.36
	**JoLO**	11.5 (2.8)	11.4 (2.0)	0.82
	**HVLT-R Total Recall**	19.9 (6.8)	22.8 (5.4)	0.14
	**HVLT- Delayed Recall**	6.4 (3.3)	8.3 (3.6)	0.10
	**Semantic**	19 (7)	17 (8)	0.40
**Gait**	**Pace & Turning**	0.22 (1.10)	0.30 (0.76)	0.76
	**Variability**	0.42 (0.87)	0.53 (1.17)	0.74
	**Rhythm**	0.33 (1.68)	0.23 (1.01)	0.81
	**Trunk**	0.02 (1.10)	-0.12 (0.72)	0.65
**Balance**	**Sway Area & Jerk**	0.36 (1.40)	0.31 (1.22)	0.92
	**Velocity**	0.07 (0.85)	0.22 (0.86)	0.64
	**Frequency ML**	0.20 (1.06)	-0.29 (1.17)	0.16
	**Frequency AP**	0.15 (1.17)	-0.17 (0.67)	0.38

Abbreviations: MDS-UPDRS III (Movement Disorders Society Unified Parkinson's disease Rating Scale), LEDD (Levodopa Equivalent Daily Dose), NCI (no cognitive impairment), MCI (mild cognitive impairment) and PDD (Parkinson's disease dementia), MoCA (Montreal Cognitive Assessment), LNST (Letter Number Sequencing Test), TMT (Trial Making Test), JoLO (Judgement of

Line Orientation), HVLT-R (Hopkins Verbal Learning Test-Revised), ML (medio-lateral) and AP (anterior-posterior). Gait and balance data provided are domain scores.

Supplementary Table 3. Gait and balance characteristics between those with and without GBA variants. Values in table represent Mean (SD).

		No GBA Variant (N=289)	GBA Variant (N=43)
		Mean (SD)	Mean (SD)
**Gait**	**Pace and Turning**		
	Gait Speed (m/s)	0.99 (0.19)	0.89 (0.22)
	Stride Length (m)	1.11 (0.18)	1.03 (0.21)
	Foot Strike Angle (deg)	19.11 (6.05)	17.56 (7.36)
	Turn Duration (s)	2.48 (0.41)	2.58 (0.51)
	Turn Velocity (deg/s)	152.93 (33.66)	143.27 (38.52)
	Steps per Turn (#)	4.13 (0.80)	4.19 (0.97)
	**Rhythm**		
	Stride Time (s)	1.14 (0.10)	1.18 (0.17)
	Stance Time (s)	0.69 (0.08)	0.73 (0.12)
	Swing Time (s)	0.44 (0.04)	0.45 (0.05)
	**Variability**		
	Stride Length SD (m)	0.06 (0.02)	0.07 (0.03)
	Foot Strike Angle SD (deg)	2.48 (0.88)	2.97 (1.04)
	Stride Time SD (s)	0.04 (0.02)	0.05 (0.03)
	**Trunk Movement**		
	ROM Coronal (deg)	4.61 (1.97)	4.57 (2.34)
	ROM Sagittal plane (deg)	4.30 (1.29)	4.31 (1.48)
	ROM Transverse plane (deg)	8.39 (3.12)	7.88 (3.35)
**Balance**	**Sway Area & Jerkiness**		
	Sway Area	0.010 (0.025)	0.060 (0.176)
	Jerk AP (m^2/s^5)	0.006 (0.011)	0.059 (0.241)
	Jerk ML(m^2/s^5)	0.004 (0.023)	0.050 (0.170)
	RMS AP (m/s^2)	0.09 (0.06)	0.14 (0.15)
	RMS ML (m/s^2)	0.04 (0.03)	0.07 (0.10)
	**Sway Velocity**		
	Velocity AP (m/s)	0.35 (0.31)	0.44 (0.42)
	Velocity ML (m/s)	0.15 (0.13)	0.22 (0.24)
	**Sway Frequency ML**		
	Frequency ML (Hz)	0.79 (0.34)	0.88 (0.45)
	95 Frequency ML (Hz)	2.30 (0.53)	2.39 (0.56)
	Cent Frequency ML (Hz)	0.98 (0.27)	1.06 (0.34)
	**Sway Frequency AP**		
	Frequency AP (Hz)	0.53 (0.20)	0.60 (0.30)
	95 Frequency AP (Hz)	1.59 (0.42)	1.72 (0.50)
	Cent Frequency AP (Hz)	0.66 (0.18)	0.74 (0.25)

‡Analysis adjusted for age, gender, disease duration, testing site and APOE Ɛ4 group status. † Values log transformed for statistical analysis. Abbreviations: SD (standard deviation), ROM (range of movement), AP (anterior-posterior), ML (medio-lateral), RMS (root mean square).

Supplementary Table 4. Gait and balance domains between those with and without GBA variants controlling for cognitive group. Values in table represent Mean (SD).

		No GBA Variant (N=289)	GBA Variant (N=43)	Unadjusted Differences, Standardized			Adjusted for cognitive group, Standardized[Ͳ]			
		Mean (SD)	Mean (SD)	β	*p*	95% CI	β	β	*p*	95% CI
**Gait**	Pace & Turning	0.03 (0.82)	-0.25 (0.93)	-0.28	0.069	-0.58, 0.02	**-0.38**	-0.26	0.075	-0.54, 0.03
	Rhythm	-0.04 (0.87)	0.28 (1.37)	0.32	0.151	-0.12, 0.75	0.34	0.37	0.093	-0.06, 0.80
	Variability	-0.07 (0.79)	0.47 (1.02)	**0.54**	**0.001**	0.21, 0.87	**0.53**	**0.49**	**0.005**	0.15, 0.83
	Trunk Movement	0.01 (0.74)	-0.05 (0.92)	-0.06	0.699	-0.35, 0.24	-0.09	-0.01	0.944	-0.30, 0.28
**Balance**	Sway Area & Jerk†	-0.06 (0.82)	0.43 (1.33)	**0.50**	**0.017**	0.09, 0.91	**0.50**	**0.46**	**0.019**	0.08, 0.84
	Sway Velocity†	-0.04 (0.80)	0.24 (0.91)	0.27	0.060	-0.01, 0.56	**0.28**	0.25	0.073	-0.02, 0.52
	Sway Frequency ML	-0.03 (0.89)	0.19 (1.09)	0.22	0.204	-0.12, 0.56	0.22	0.16	0.398	-0.21, 0.53
	Sway Frequency AP	-0.05 (0.86)	0.31 (1.22)	0.36	0.063	-0.02, 0.73	0.37	0.34	0.083	-0.04, 0.72

† Values log transformed for statistical analysis; mean and SD given on original scale.

ͲAnalysis adjusted for age, gender, disease duration, testing site, APOE Ɛ4 group status and cognitive group

Supplementary Table 5. Cognitive, gait and balance characteristics comparing mild and severe GBA variants

		**Mild (N=11)**	**Severe (N=10)***	***p*** ^{T}
**Cognition**	**MoCA**	26.2 (3.0)	23.0 (6.2)	(.32)
	**TMT B-A**	51 (24)	96 (91)	(.71)
	**LNST**	10.3 (3.0)	9.2 (3.7)	(.47)
	**JoLO**	11.2 (2.9)	12.0 (2.7)	(.45)
	**HVLT-R Total Recall**	22.5 (4.7)	23.2 (6.4)	(.76)
	**HVLT- Delayed Recall**	7.9 (3.7)	8.7 (3.5)	(.70)
	**Semantic**	18 (7)	20 (8)	(.52)
**Gait**	**Pace & Turning**	-0.20 (0.56)	-0.24 (1.10)	(.73)
	**Variability**	0.50 (0.61)	0.32 (1.14)	(.34)
	**Rhythm**	-0.05 (0.89)	0.79 (2.31)	(.91)
	**Trunk**	-0.001 (1.38)	0.04 (0.68)	(.27)
**Balance**	**Sway Area & Jerk**	-0.42 (1.47)	0.07 (1.12)	(.43)
	**Velocity**	-0.12 (0.90)	0.28 (0.92)	(.38)
	**Frequency ML**	0.51 (1.39)	0.37 (1.17)	(.91)
	**Frequency AP**	0.31 (1.32)	0.58 (1.60)	(.68)

*comprised of severe and null mutation severity

^{T} Mann-Whitney U Test
